# Passing Down Pollution: Calculating Intergenerational Exposure to PCBs

**DOI:** 10.1289/ehp.119-a219a

**Published:** 2011-05

**Authors:** Rebecca Kessler

**Affiliations:** **Rebecca Kessler**, based in Providence, RI, writes about science and the environment for various publications. She is a member of the National Association of Science Writers and the Society of Environmental Journalists

Countries around the world began phasing out production of polychlorinated biphenyls (PCBs) in the 1970s after adverse health and environmental effects of these chemicals came to light. A new study calculating intergenerational differences in human PCB exposure now suggests some of the highest exposures occurred well after the chemicals were phased out **[*EHP*** **119(5):641–646; Quinn et al.]**.

PCBs are manufactured organic chemicals that were used in numerous industrial applications starting in the 1920s. Although they have been phased out of production, PCBs are still being released into the environment from preexisting applications and improper disposal of products that contain them. Because of their chemical stability and transportability they bioaccumulate in the food chain, readily enter the human food supply, and pass from mother to infant *in utero* and during breastfeeding, with potentially harmful effects on the endocrine, immune, nervous, and reproductive systems.

In 2010 three authors of the current study and another colleague developed and tested a mechanistic model called CoZMoMAN that accounts for PCBs’ emissions history and movement through the environment, the food chain, and the human body to predict concentrations in a population’s body fat. For the present study, the authors used CoZMoMAN to calculate how PCB concentrations in fat changed over the lifetimes of hypothetical Swedish women born each decade between 1920 and 2010. They also analyzed how such factors as the age at which a woman first gives birth, the number of children she bears (parity), and whether she breastfeeds her babies affect both her PCB body burden and that of her children.

The model predicted that a woman’s PCB exposure was determined primarily by when she was born. Women born in the 1960s were predicted to have the highest cumulative lifetime exposure—PCBs were already in widespread use when these women were born, and emissions peaked as they were maturing in the 1970s. However, it was women born in the 1980s who experienced the highest prenatal exposure and the highest peak concentration at any given age. Prenatal exposure to PCBs can be associated with serious neurologic and reproductive consequences.

Prenatal exposure was strongly influenced by children’s birth order, whereas postnatal exposure was influenced by whether children were fed breast milk or infant formula. A mother’s reproductive characteristics—specifically breastfeeding and parity—had a greater impact on her children’s PCB burden than on her own, but these characteristics only appeared to matter for infants born after the PCB phaseout. Because both pre- and postnatal PCB exposures have been associated with health complications, the results suggest the health effects of PCBs are likely to persist over several more generations.

